# Caliper verification and gap measurements of kinematic alignment total knee arthroplasty utilizing an imageless, accelerometer-based navigation system

**DOI:** 10.1186/s43019-025-00260-x

**Published:** 2025-02-17

**Authors:** James H. Sikes, Drew P. Melancon, Isaac J. Spears, Evan H. Powers, Spencer J. Montgomery

**Affiliations:** University of Mississippi Medical Center, 2500 N State St, Jackson, MS 39216 USA

**Keywords:** Kinematic alignment, Total knee arthroplasty, Total knee replacement, Caliper, Navigation

## Abstract

**Purpose:**

Kinematic alignment (KA) in total knee arthroplasty (TKA) aims to restore the patient’s knee to the prearthritic state. The purpose of this study was to investigate the accuracy of using an implant-agnostic, imageless, accelerometer-based navigation system to perform KA TKA on the basis of caliper verification and quantification of the flexion and extension gaps.

**Materials and methods:**

Seven cadaveric lower extremities underwent primary TKA utilizing a kinematic alignment workflow with the imageless navigation system. Accuracy of the technique was confirmed through caliper verification of bone cuts.

**Results:**

All cuts were within 1 mm of anticipated measurements, except for the lateral tibial fragment, which averaged 1 mm (standard deviation [SD] 0.9 mm) thicker than anticipated. In extension, medial and lateral gaps were symmetric and averaged within 0.6 mm of expectation. In flexion, the medial gap averaged within 0.5 mm of expectation, while the lateral gap averaged 2.6 mm larger than the symmetric expectation, consistently producing a trapezoidal space.

**Conclusions:**

The implementation of an accelerometer-based navigation system in KA TKA allows for highly accurate results, which was confirmed with caliper verification. This workflow produced a symmetric extension gap and a trapezoidal flexion gap with an average increased lateral flexion gap of 2.6 mm compared with the medial side.

## Introduction

The rate of total knee arthroplasty (TKA) in the USA is steadily increasing, and the volume of TKA is expected to increase by 24% for each 5-year period through 2040 [1]. Historically, mechanical alignment (MA) TKA involved perpendicular (to the mechanical axis) distal femoral and proximal tibial resections. However, given the unique anatomy and alignment of each patient, the standardization of implant alignment seen in MA TKA requires the release of ligaments and other soft tissue elements about the knee to achieve a well-balanced TKA. Instability due to incorrect balancing of ligaments or incorrect bone cuts is one of the leading causes of clinical failure in primary TKA [2].

Kinematic alignment (KA) in TKA is based on performing patient-specific bony resections, accounting for cartilage loss when required and the thickness of the saw blade so that the thickness of the implant will restore the knee to the prearthritic state [3]. This approach is suggested to provide the patient with a naturally well-balanced knee without the need for soft tissue releases [4]. Kinematic alignment has been shown in multiple studies to improve patient functional outcomes without increased complications [5–8]. Furthermore, across all coronal plane alignment of the knee (CPAK) types, KA TKA has been shown to produce superior gap balance compared with MA TKA, most significantly in CPAK types I,II, and IV [9].

While the original KA TKA technique is based on the use of manual instrumentation, caliper measurements may not be reliable in all conditions, such as acquired deformity or post-traumatic cartilage or bone loss [10]. Previously, Hutt et al. have demonstrated the ability to perform KA TKA with an optical navigation system utilizing mechanical lateral distal femoral angle (mLDFA) and mechanical medial proximal tibial angle (mMPTA) measurements, with promising clinical results [11]. Previous research has analyzed the use of accelerometer-based navigation platforms in TKA; however, this was not in accordance with KA principles [12–14]. The goal of this study was to perform KA TKA with an imageless, accelerometer-based navigation system utilizing radiographic mLDFA and mMPTA angles and to provide caliper measurements of resections for validation and to ensure the results were comparable to the classically described technique. In addition, we sought to objectively evaluate the gap measurements of the flexion and extension gaps, both medially and laterally, after KA TKA, utilizing a digital tensioning device.

## Methods and materials

### Subjects

A cadaveric surgical procedural study was conducted. This study utilized four specimens to carry out bilateral primary TKA using kinematic alignment principles. The first lower extremity was excluded from the study, as it served to establish the standardized workflow and equipment requirements that would be utilized in remainder of the specimens. In total, seven lower extremity knees (four left knees and three right knees) underwent TKA.

All specimens were in the sixth to eighth decade of life without any prior surgeries or known injuries to the knees. Inclusion criteria included adult male or female specimens with intact bilateral lower extremities. Body habitus and lower extremity alignment was not considered as part of the inclusion criteria. Cause of death did not exclude specimens from this study. Exclusion criteria ensured specimens did not have absent lower extremity anatomy, any previous lower extremity surgery, traumatic distortion, or inadequate preservation affecting the anatomy of the lower extremities. All specimens were preserved in a low-temperature environment. No specimens were excluded from the study.

### Outcome parameters

Preoperative X-rays were utilized to identify the arithmetic hip–knee–ankle angle (aHKA), mechanical lateral distal femoral angle (mLDFA), mechanical medial proximal tibial angle (mMPTA), and joint line obliquity (JLO). A standard caliper was used to record the thickness of bone fragments (medial distal femoral, lateral distal femoral, medial posterior femoral, lateral posterior femoral, medial tibial, and lateral tibial). The primary outcome assessed was the accuracy of the bony resections and the subsequent accuracy of the gap compared with the anticipated and predicted gap. The “anticipated cuts” were based on the set resection depths and accounted for 1 mm bone loss owing to the kerf of the saw blade. Cartilage was not cleared down to subchondral bone on any specimens; a correction of 1 mm was applied to fragments with “partial wear” and 2 mm applied to cuts with “full thickness cartilage loss.” The “anticipated gaps” were determined on the basis of the summation of the bone cut measurements and an anticipated joint space of 1 mm and did not account for any potential asymmetry in the soft tissue laxity. Similarly, the “predicted gap” was based on a femoral implant thickness of 9 mm, a tibial implant thickness of 10 mm, and 1 mm remaining joint space in the medial extension, lateral extension, and medial flexion gaps, resulting in an predicted gap of 20 mm. However, the predicted joint space of the lateral flexion gap was estimated to be 3 mm, giving a predicted gap of 22 mm on the basis of previous studies [15–17].

### Surgical technique

**Fig. 1:**
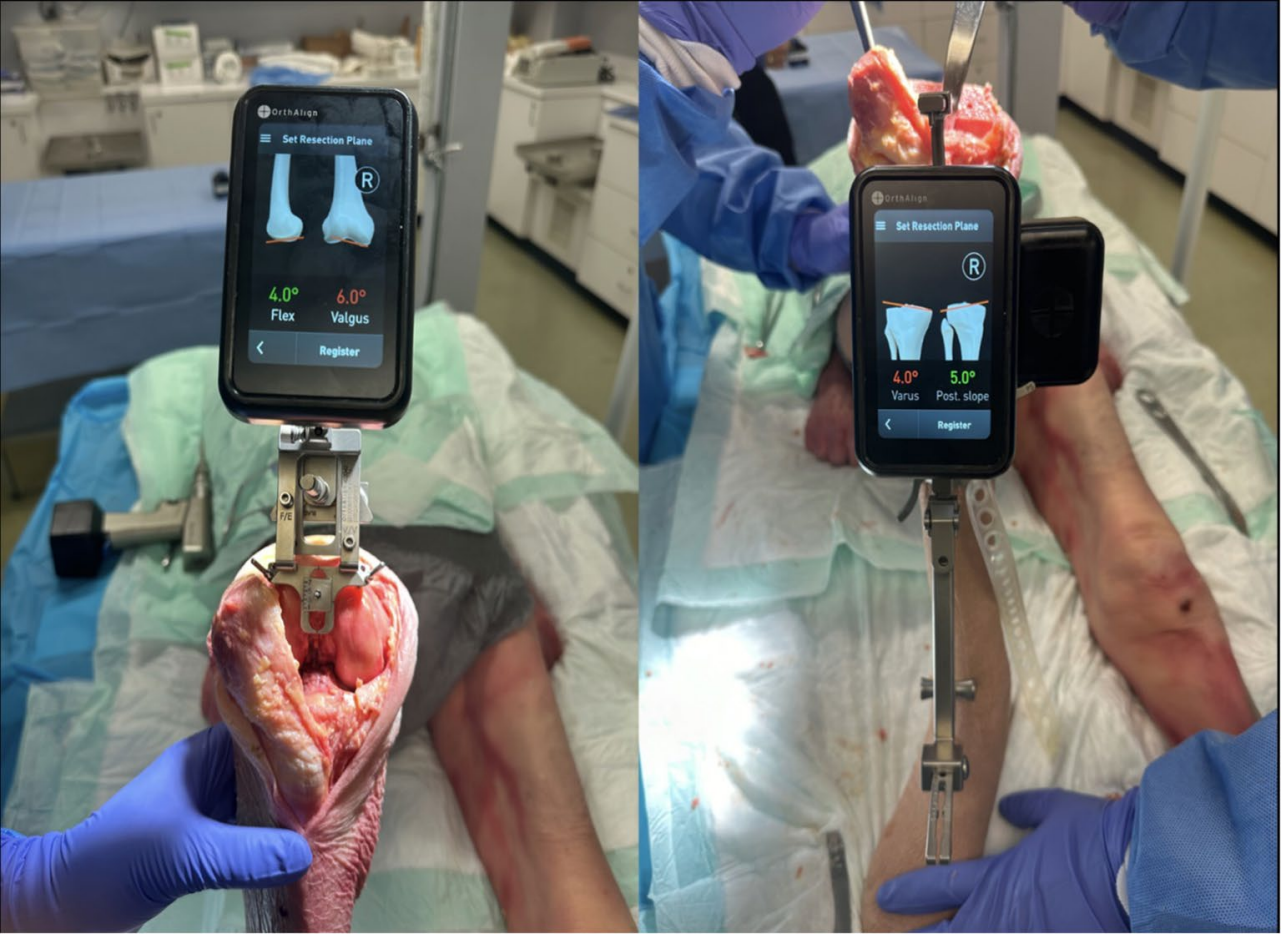
The OrthAlign settings demonstrate femoral flexion (degrees) and tibial slope (degrees)

All procedures were completed by the senior author, and all data were collected over a 4-week period. The implant-agnostic accelerometer-based navigation system (OrthAlign^®^, Irvine, CA, USA) was employed to guide necessary bony cuts and soft tissue balancing. Preoperative X-rays were obtained for each lower extremity, and the mechanical axis was referenced to obtain the mLDFA and mMPTA, which were utilized for setting the femoral valgus angle and tibial varus angle, respectively. Femoral flexion was set to 4° for all cases, and tibial slope was set to 5° for all cases (Fig. 1). A medial parapatellar approach was utilized to expose the tibiofemoral joint space. Any signs of arthritic or degenerative changes were noted before proceeding.

**Fig. 2:**
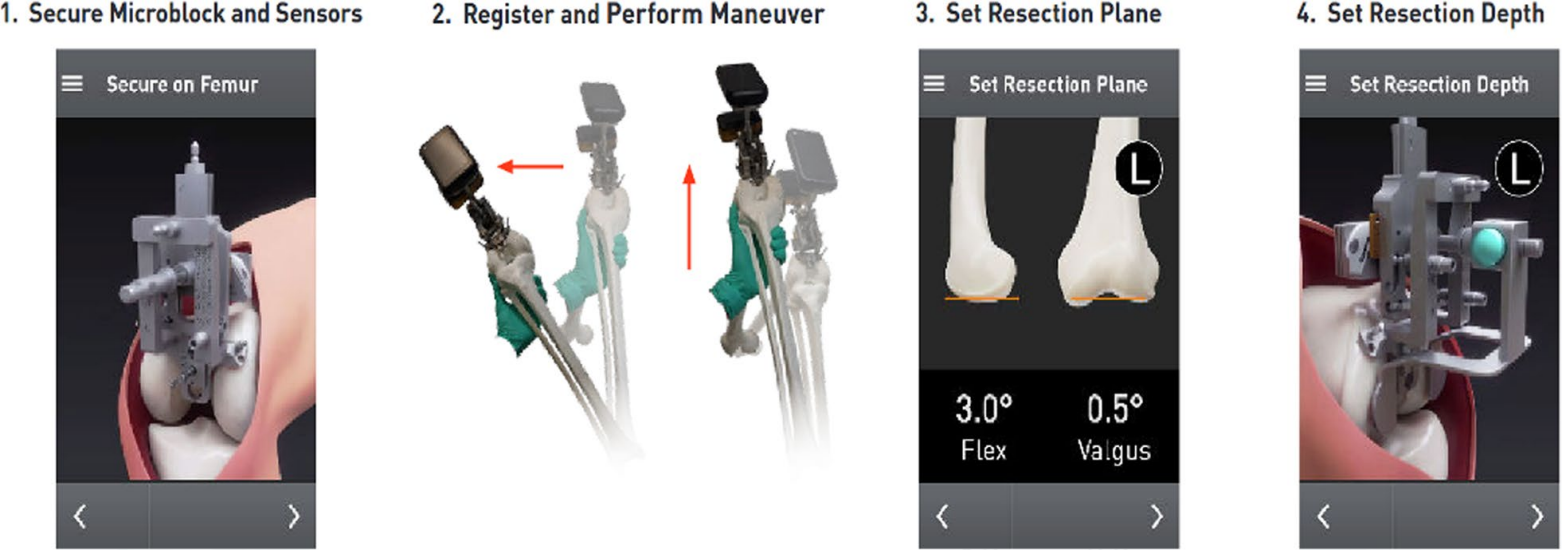
The femur is registered, and resections are made on the basis of desired settings

**Fig. 3:**
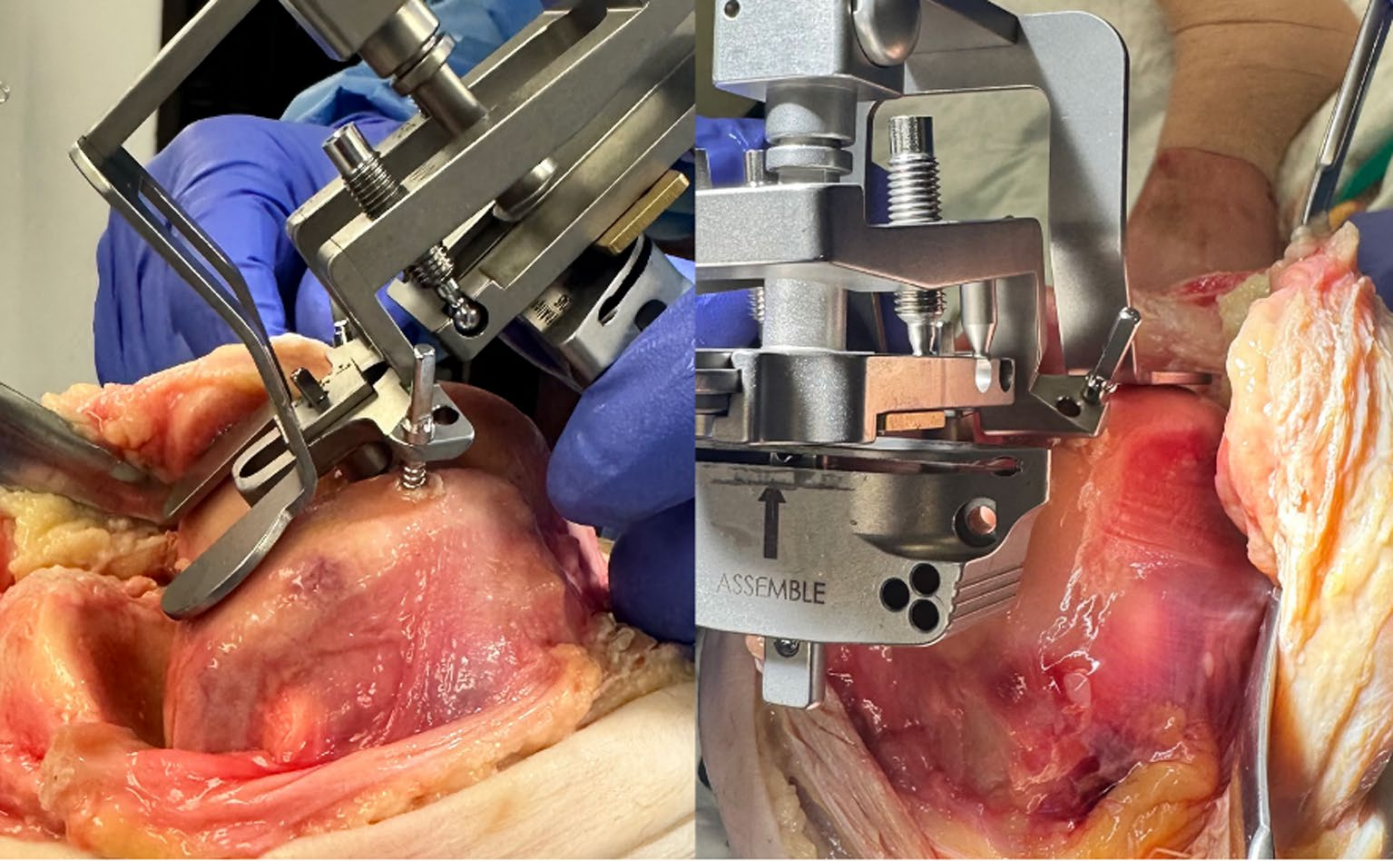
Depth resection paddles are resting on healthy cartilage on the medial and femoral condyles when placed according to mLDFA measurement

After arthrotomy, a medial release was performed to the mid-sagittal line, the anterior horns of the medial and lateral menisci were removed, and the anterior cruciate ligament was resected; the posterior cruciate ligament was retained in all cases. A headless pin was drilled to the center of the femoral head so that the positioning of the femur could be registered (Fig. 2). The microblock was then secured to the femur while the reference sensor and Lantern^®^ unit were then mounted. The knee was placed at 90° of flexion and maneuvered following on-screen prompts to register the location of the center of the femoral head. The distal femur resection plane was set according to the templated angles. The depth of resection for the distal femur was manually set to 9 mm to replicate the thickness of implant being used (Enovis Empowr 3D, Wilmington, DE, USA). The resection guide was lowered toward the bone until it came to rest on healthy cartilage (Fig. 3), the cutting block was pinned to the anterior femur, the distal guide was removed, and the surgeon proceeded with the distal femur resection. The thickness of the distal femoral fragments was recorded using a caliper (medial distal and lateral distal). The posterior femoral condyles were then resected utilizing the 4-in-1 guide from the implant manufacturer, setting the rotation at 0° to the posterior condylar axis. The posterior condylar bone cuts were then measured with a caliper, with the expectation of 8 mm resection plus 1 mm for thickness of the sawblade, as the posterior condylar thickness of the Enovis Empowr 3D femoral implant is also 9 mm.

**Fig. 4:**
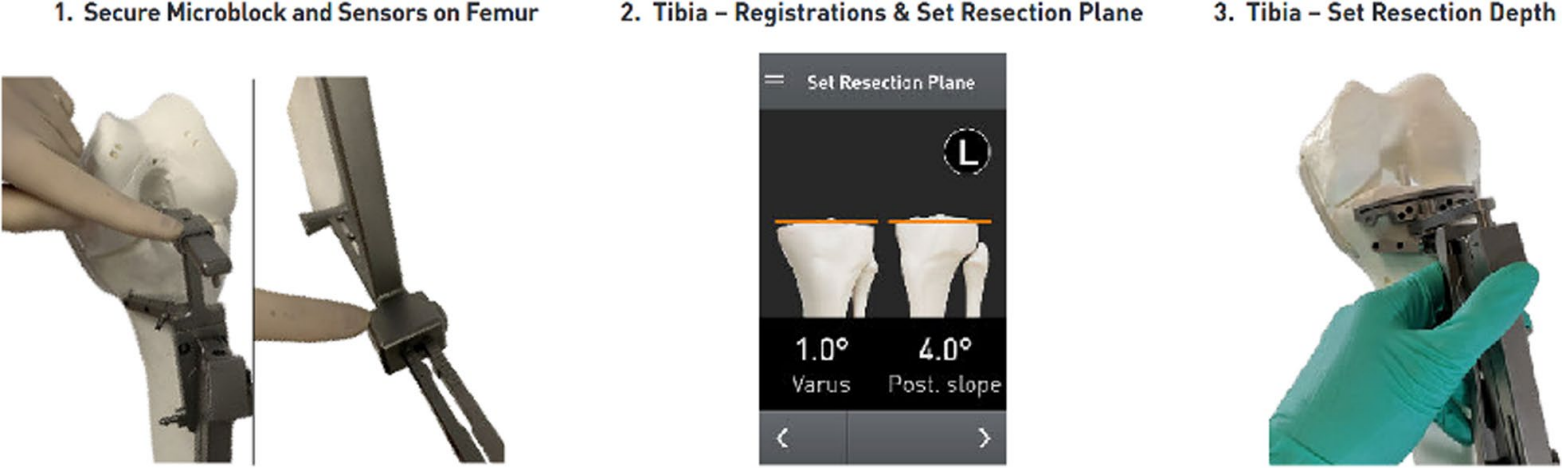
The tibia is registered, and resections are made on the basis of desired settings

**Fig. 5:**
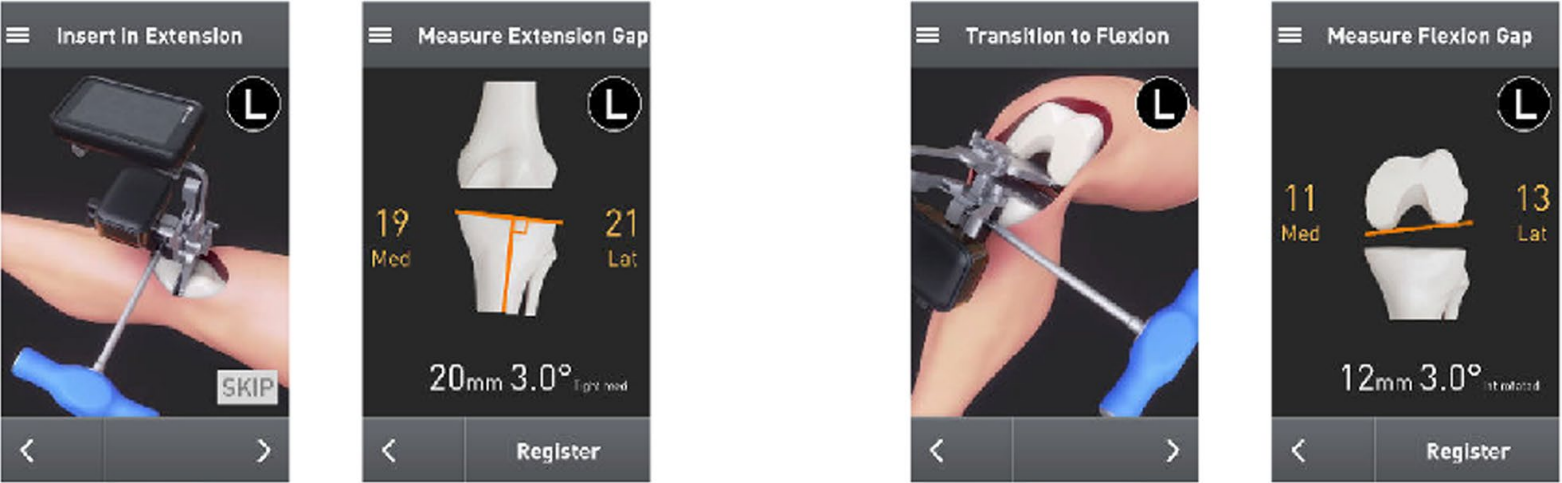
The gap measurement was obtained by inserting the paddles and turning the torque driver

**Fig. 6:**
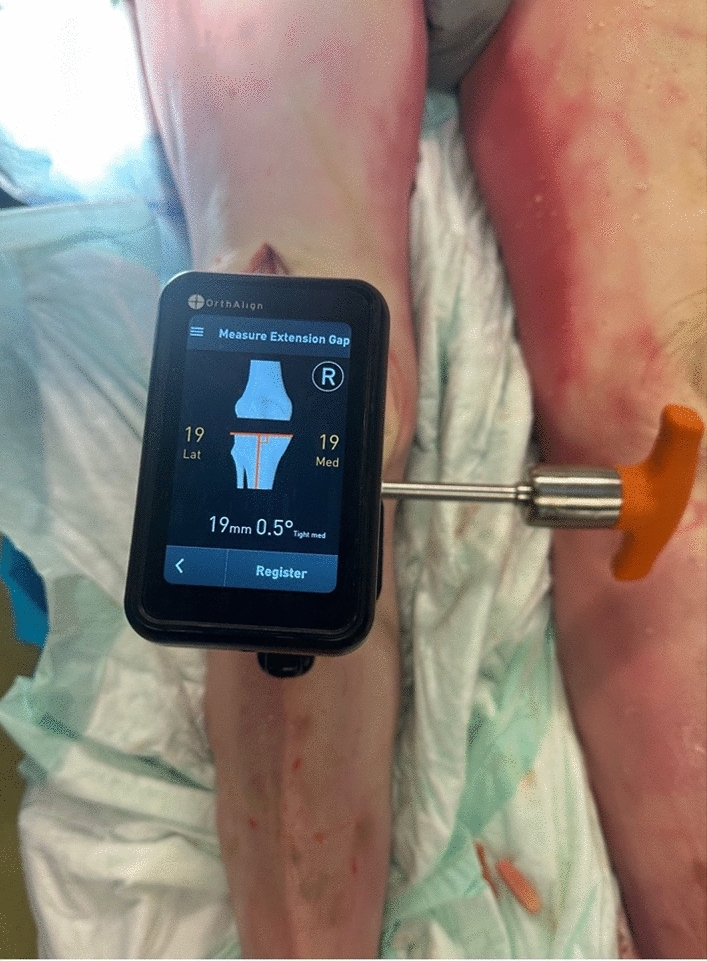
Gap assessment was performed in extension, demonstrating a symmetric extension gap

Next, the surgeon proceeded to register the tibia for resection (Fig. 4). The midline probe was positioned at the insertion of the anterior cruciate ligament on the tibia. The tibial offset was set, and then, both malleoli were registered using the malleolar probe. The tibial resection plane was then set by adjusting the tibial coronal alignment to the preoperative templated angle and setting posterior slope to 5°. The resection depth was set to 8 mm and positioned in the middle of the articular surface, and the tibial jig was pinned in placed. Medial and lateral tibial fragment thickness was recorded after resection. No further adjustments or cuts were performed. The paddles of the gap-measuring device were inserted into the joint space, and a torque driver was rotated clockwise, expanding the paddles until an audible click was heard; the gap measurement was collected and registered (Fig. 5). The proximal paddle rotates about a central axis to provide independent readings for medial and lateral sides. Gap balance measurements were obtained in flexion and extension gaps using the OrthAlign^®^ Lantern^®^ device (Fig. 6). The tensioning device was tightened to 30 in-lb of force through a torque driver, which produces between 250–300 N between the measurement paddles (OrthAlign, data on file). No ligament balancing or releases were performed.

### Statistical analysis

SPSS version 29 was used to carry out necessary statistical analysis. All preoperative data regarding the status of the joint were recorded (Table 1). Paired samples *t*-tests were used as the statistical methodology, as the data in this study were continuous, and each data point was paired to another related measurement. The team of investigators was interested in determining whether the two measurements were significantly different. A paired samples *t*-test was employed to determine whether the difference between the anticipated and calipered cuts was significant using *p* < 0.05 (Table 2). A paired samples *t*-test was used to compare the anticipated gap and the measured gap, as well as the predicted gap and the measured gap, using *p* < 0.05 (Table 4). Mean and standard deviation were obtained for bone resections (Table 1), the accuracy of these resections (Table 2), and gap measurements with the subsequent accuracy (Table 3). Data are expressed as mean (SD).

**Table 1:** Radiographic measurements, planned navigation inputs, and caliper measurements of bony resections for each of the seven specimens

Specimen number + side	1 left	2 left	2 right	3 left	3 right	4 left	4 right
	Caliper measurements (mm)						
mLDFA (°)	88.0	86.5	86.5	84.0	84.0	89.0	88.0
mMPTA (°)	86.0	86.0	86.0	86.0	86.0	86.0	85.0
Femoral valgus (°)	2.0	3.5	3.0	6.0	6.0	1.0	2.0
Femoral flexion (°)	3.0	3.5	3.0	4.0	4.0	4.0	4.0
Tibial varus (°)	4.0	3.5	4.0	4.0	4.0	4.0	5.0
Tibial slope (°)	5.0	5.0	5.0	5.0	5.0	5.0	5.0
Arithmetic HKA	−2.0	−0.5	−0.5	2.0	2.0	−3.0	−3.0
JLO	174.0	172.5	172.5	170.0	170.0	175.0	173.0
CPAK	2	2	2	2	2	1	1
Medial distal femur (mm)	7.0	8.0	9.0	7.0	7.0	8.0	7.0
Lateral distal femur (mm)	7.0	8.0	9.0	7.0	7.0	8.0	7.0
Medial posterior femur (mm)	8.0	9.0	8.0	7.0	8.0	7.0	8.0
Lateral posterior femur (mm)	8.0	9.0	8.0	8.0	8.0	7.0	8.0
Medial tibia (mm)	8.0	8.0	8.0	8.0	10.0	7.0	7.0
Lateral tibia (mm)	8.0	9.0	10.0	8.0	10.0	9.0	9.0

**Table 2:** Cartilage status, anticipated and measured bony resection values (mm), and resultant accuracy of resections

Specimen number		1 left	2 left	2 right	3 left	3 right	4 left	4 right	Mean (SD) *p*-Value
Distal medial	Status	Unworn	Unworn	Unworn	Partial wear	Unworn	Unworn	Unworn	0.28 (0.76) *p* = 0.356
	Anticipated cut	8.0	8.0	8.0	7.0	8.0	8.0	8.0	
	Calipered cut	7.0	8.0	9.0	7.0	7.0	8.0	7.0	
	Accuracy	1	0	−1	0	1	0	1	
Distal lateral	Status	Unworn	Unworn	Unworn	Unworn	Unworn	Unworn	Unworn	0.43 (0.79) *p* = 0.200
	Anticipated cut	8.0	8.0	8.0	8.0	8.0	8.0	8.0	
	Calipered cut	7.0	8.0	9.0	7.0	7.0	8.0	7.0	
	Accuracy	1	0	−1	1	1	0	1	
Posteromedial	Status	Unworn	Unworn	Unworn	Partial wear	Unworn	Partial wear	Unworn	−0.14 (0.69) *p* = 0.604
	Anticipated cut	8.0	8.0	8.0	7.0	8.0	8.0	8.0	
	Calipered cut	8.0	9.0	8.0	8.0	8.0	7.0	8.0	
	Accuracy	0	−1	0	−1	0	1	0	
Posterolateral	Status	Unworn	Unworn	Unworn	Unworn	Unworn	Unworn	Unworn	0.00 (0.57) *p* = 1.000
	Anticipated cut	8.0	8.0	8.0	8.0	8.0	8.0	8.0	
	Calipered cut	8.0	9.0	8.0	8.0	8.0	7.0	8.0	
	Accuracy	0	−1	0	0	0	1	0	
Medial tibia	Status	Unworn	Partial wear	Partial wear	Unworn	Unworn	Partial wear	Partial wear	−0.14 (0.90) *p* = 0.689
	Anticipated cut	9.0	7.0	7.0	9.0	9.0	7.0	7.0	
	Calipered cut	8.0	8.0	8.0	8.0	10.0	7.0	7.0	
	Accuracy	1	−1	−1	1	−1	0	0	
Lateral tibia	Status	Unworn	Unworn	Unworn	Unworn	Unworn	Unworn	Unworn	−1.00 (0.82) *p* = 0.018
	Anticipated cut	8.0	8.0	8.0	8.0	8.0	8.0	8.0	
	Calipered cut	8.0	9.0	10.0	8.0	10.0	9.0	9.0	
	Accuracy	0	−1	−2	0	−2	−1	−1

**Table 3:** Assessment of gap balance comparing the anticipated gap, measured gap, and predicted gap in reference to the corresponding femoral bony resection (mm)

Specimen number		1 left	2 left	2 right	3 left	3 right	4 left	4 right	Mean	SD
Medial extension gap	Anticipated gap	18.0	20.0	21.0	17.0	20.0	19.0	18.0	19.00	1.41
	Measured gap	19.0	19.0	19.0	19.0	21.0	20.0	19.0	19.43	0.79
	Accuracy	−1	1	2	−2	−1	−1	−1	−0.43	1.40
	Actual gap versus predicted gap (20 mm)	−1	−1	−1	−1	1	0	−1	−0.57	0.79
Lateral extension gap	Anticipated gap	17.0	19.0	21.0	17.0	19.0	19.0	18.0	18.57	1.40
	Measured gap	19.0	19.0	19.0	19.0	21.0	20.0	19.0	19.43	0.79
	Accuracy	−2	0	2	−2	−2	−1	−1	−0.86	1.46
	Actual gap versus predicted gap (20 mm)	−1	−1	−1	−1	1	0	−1	−0.57	0.79
Medial flexion gap	Anticipated gap	18.0	20.0	19.0	19.0	20.0	18.0	18.0	18.86	0.90
	Measured gap	20.0	19.0	19.0	19.0	21.0	20.0	21.0	19.86	0.90
	Accuracy	−2	1	0	0	−1	−2	−3	−1.00	1.41
	Actual gap versus predicted gap (20 mm)	0	−1	−1	−1	1	0	1	−0.14	0.90
Lateral flexion gap	Anticipated gap	18.0	20.0	20.0	18.0	20.0	18.0	19.0	19.00	1.00
	Measured gap	20.0	19.0	23.0	21.0	23.0	24.0	21.0	21.57	1.81
	Accuracy	−2	1	−3	−3	−3	−6	−2	−2.57	2.07
	Actual gap versus predicted gap (22 mm)	−2	−3	1	−1	1	2	−1	−0.43	1.81

## Results

Five lower extremities were CPAK classification 2, and two lower extremities were CPAK classification 1. Preoperative radiographic measurements, planned resection values, and caliper verification values are presented in Table 1.

The mean accuracy, standard deviation, and *p*-value for the difference between the anticipated and calipered cut were obtained (Table 2). Mean distal medial resection was 7.57 mm (SD 0.8 mm), distal lateral was 7.6 mm (SD 0.8 mm), medial flexion was 7.86 mm (SD 0.69 mm), posterior lateral was 8.00 mm (SD 0.6 mm), medial tibia was 8.00 mm (SD 1.00 mm), lateral tibia was 9.00 mm (SD 0.9 mm).

The anticipated, measured, and predicted gaps are summarized in Table 3. The mean anticipated and measured gaps, along with the accuracy (mean difference), were as follows: medial extension gap 19.0 mm (SD 1.41 mm), 19.43 mm (SD 0.79 mm), and −0.43 mm (SD 1.40 mm); lateral extension gap 18.57 mm (SD 1.40 mm), 19.43 mm (SD 0.79 mm), and −0.86 mm (SD 1.46 mm); medial flexion gap 18.86 mm (SD 0.90 mm), 19.86 mm (SD 0.90 mm), and −1.0 mm (SD 1.41 mm); and lateral flexion gap 19.0 mm (SD 1.0 mm), 21.57 mm (SD 1.81 mm), and −2.57 mm (SD 2.07 mm), respectively. When compared with the a priori established predicted gap measurements, the actual gap measurements differed by −0.57 mm (SD 0.79 mm) in both the medial and lateral extension gaps, −0.14 mm (SD 0.90 mm) in the medial flexion gap, and −0.43 mm (SD 1.81 mm) in the lateral flexion gap.

Last, the difference between the measured gap and the anticipated or ideal gaps were assessed with an independent samples *t*-test to determine whether there was statistical significance (Table 4). The difference between the measured gap and the anticipated gaps was insignificant for the medial extension, lateral extension, and medial flexion gaps, with *p* = 0.448, *p* = 0.172, and *p* = 0.111, respectively (Table 4). The only gap that was found to be significantly different from than the anticipated gap was the lateral flexion gap (*p* = 0.017). The difference between the measured gaps and the predicted gaps were all found to be insignificant.

**Table 4:** Differences between anticipated, measured, and predicted gaps, with significance noted when *p* < 0.05 in bold print

Medial extension gap	Anticipated gap versus measured gap Predicted gap versus measured gap	*p* = 0.448 *p* = 0.103
Lateral extension gap	Anticipated gap versus measured gap Predicted gap versus measured gap	*p* = 0.172 *p* = 0.103
Medial flexion gap	Anticipated gap versus measured gap Predicted gap versus measured gap	*p* = 0.111 *p* = 0.689
Lateral flexion gap	Anticipated gap versus measured gap Predicted gap versus measured gap	***p* = 0.017** *p* = 0.555

## Discussion

This study showed that an imageless, accelerometer-based navigation system can be used to perform KA TKA with a high degree of accuracy, suggesting that the implementation of imageless navigation on the basis of coronal mMPTA and mLDFA measurements is a reliable approach. Resections in this study were found to deviate from the expected measurement by less than 0.5 mm, with the exception of the lateral tibial fragment (*p* = 0.018), which averaged 1 mm of over-resection. Howell and Nedopil et al., using manual instrumentation in unrestricted caliper-verified KA TKA, found resections to be similarly accurate, within ±0.5 mm of the target [18, 19]. Li et al. found the accuracy of bony resection using robotic navigation to be within ±1.0 mm of the planned resections [20]. The caliper measurements in this study demonstrate a small and comparable deviation from expected values compared with other reported methods and would be expected to result in the same long-term success and patient satisfaction established in KA TKA patients [21, 22].

In terms of gap measurements and balance, KA TKA utilizing accelerometer-based navigation produced exceptional balance in medial and lateral compartments in extension and an expected trapezoidal flexion gap without soft tissue releases or balancing performed. The medial extension gap and lateral extension gap were symmetric at an average of 19.43 mm, the medial flexion gap was similar at 19.86 mm, and the lateral flexion gap was significantly increased at 21.57 mm, demonstrating an average increased lateral flexion laxity of 2.6 mm. This result is consistent with multiple studies that demonstrate an increased laxity in the lateral flexion space ranging from 1 to 4.7 mm in asymmetry compared with the medial flexion space [15–17, 23–25]. It has been shown that equal or increased lateral laxity in MA TKA results in improved patient-reported outcome scores after TKA [26]. In KA TKA, maintaining native asymmetric lateral flexion laxity was also shown to improve gait dynamics and range of motion and more closely matched native knee kinematics [27]. While a range of asymmetry in the flexion space is physiologic, the method of measurement is not standardized and may also influence results. In comparative studies, Nowakowski et al. applied 200 N through a digital caliper in cadaveric specimens, while Tokuhara et al. utilized the passive positioning of a patient’s knee [24, 25]. In a study by Yapp et al. assessing the use of an intraoperative pressure sensor, the recommended force for gap assessment was 5–40 lbs (22–177 N), with a maximum of 70 lb (311 N) [28]. In this study, the tensioner device produced approximately 250–300 N between the measurement paddles, which may utilize more tension than comparative studies and present a source of confounding.

There are several other limitations in our study. All specimens in this study had minimal deformity, with aHKA ranging from 2° valgus to 3° varus. The classification of partial wear in this study may not accurately describe the degree of wear that was present on the femoral or tibial resections, as it has been shown that the cartilage thickness can vary significantly, though averages approximately 2 mm [29]. Similarly, the tibial slope was set to 5° for all specimens, which could affect flexion-space measurements. A curette was not applied to the cadaveric tissues to clear off worn cartilage out of concern for potential inadvertent subchondral bone removal in the specimens. Also, long-leg radiographs (LLR) used to approximate mLDFA and mMPTA may be prone to errors owing to lower limb malrotation [30, 31]. Last, except for the lateral flexion gap, we averaged slightly tighter than the predicted gap. In this study, the tibial resection guide was set to 8 mm instead of a conventional 10 mm; this is the senior author’s preferred technique, as the tibial resection affects all gaps symmetrically, and a recut may be easily performed through the guide with slight adjustments as required. In this study, we elected to avoid recuts, as our intention was to measure the gaps produced by the initial attempted cuts, and thin recuts would have been challenging to quantify, though in clinical practice this may be required.

The described method provides an additional option for surgeons to perform KA TKA with a high degree of accuracy on the basis of mLDFA and mMPTA measurements. Specific indications where this could be beneficial include osteonecrosis of femoral condyles or the tibial plateau with collapse, absent bone or cartilage due to previous trauma, revision total knee arthroplasty, and conversion of previous unicompartmental knee arthroplasty to KA TKA.

## Conclusions

This study has shown that an imageless, accelerometer-based navigation platform can reliably assist with performing KA TKA with similar accuracy to caliper-verified conventional instrumentation, which produces a symmetric extension gap and trapezoidal flexion gap, with an average increase in the lateral flexion gap of 2.6 mm. While this is useful in primary knee arthroplasty, further studies would be warranted to investigate utilization of this navigation system for KA TKA in complex cases where caliper measurements may be unreliable or intra-articular anatomy may be distorted beyond the capabilities of conventional instrumentation.

## Data Availability

The datasets during and/or analyzed during the current study are available from the corresponding author on reasonable request.
